# Hemp Concrete with Mineral Additives as a Durable and Fire-Resistant Material in Green Construction

**DOI:** 10.3390/ma18091905

**Published:** 2025-04-23

**Authors:** Elżbieta Janowska-Renkas, Anna Król, Igor Klementowski, Michał Sokolski

**Affiliations:** 1 Department of Building Materials Engineering, Faculty of Civil Engineering and Architecture, Opole University of Technology, 45-061 Opole, Poland; 2 Department of Process and Environmental Engineering, Faculty of Mechanical Engineering, Opole University of Technology, 45-271 Opole, Poland

**Keywords:** natural materials, hemp shive, thermal conductivity, fly ash, metakaolin, durability

## Abstract

In this work, to enhance the compressive strength and evaluate the fire resistance of hemp concrete, we incorporated mineral additives such as FBC fly ash and metakaolin. This paper investigates the thermal conductivity, compressive strength, flammability, and fire resistance of hempcrete and the influence of mineral additives in the form of fly ash from fluidized bed combustion (FBC) and metakaolin on these properties. A fly ash content of 20% by weight of the binder resulted in an increase of 26% in compressive strength and about 6% in thermal conductivity compared to hemp concrete without mineral additives. The use of metakaolin in the amount of 15% by weight of the binder resulted in a 21% increase in compressive strength values with an increase in the thermal conductivity coefficient of only 0.5%. Flammability tests by direct application of a gas torch flame to the specimen surface proved the lack of flammability and spontaneous fire extinguishing ability of hempcrete. In turn, fire resistance tests showed much higher resistance to high temperatures for hempcrete modified with metakaolin, where the recorded mass loss during a 15 min test at 500 °C was ca. 58% less than in hempcrete without mineral additives, and when FBC fly ash was used, the mass loss was ca. 37% less. The obtained results are satisfactory in terms of the physico-mechanical properties of hempcrete. They also enable the replacement of traditional construction materials with waste-derived materials from other sectors of the economy, which, in the long term, will contribute to the development of green construction and support the principles of the circular economy.

## 1. Introduction

**Figure 1:**
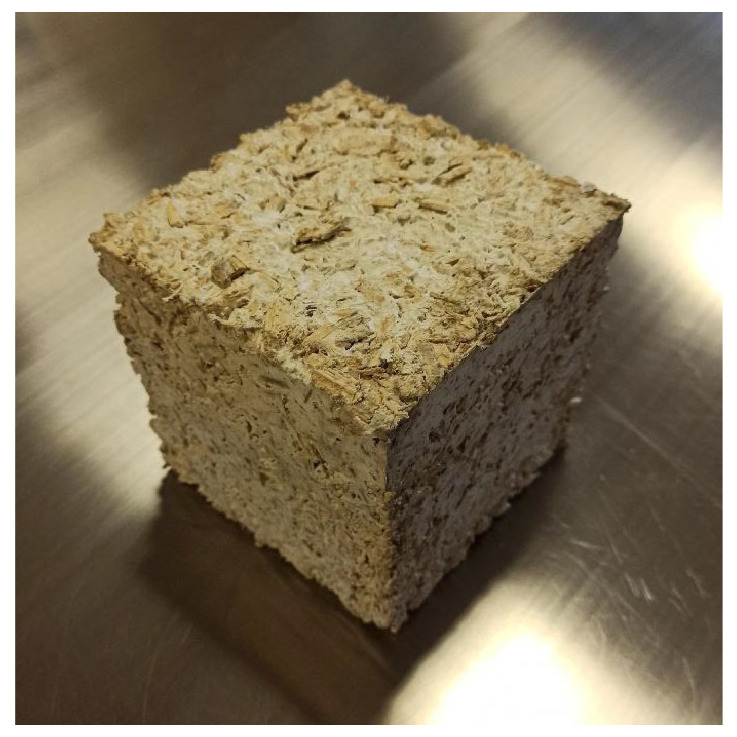
Hemp concrete sample. (Source: own photo).

**Figure 2:**
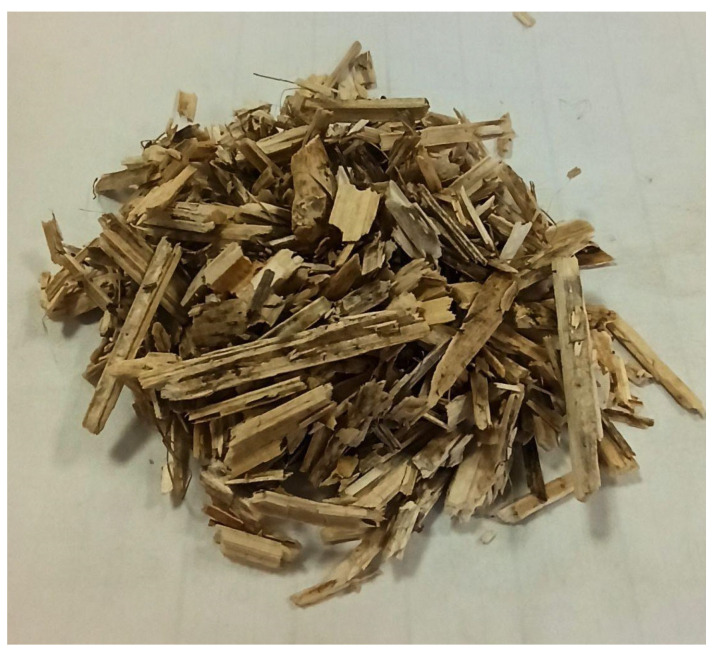
Hemp shives originating from the Bialobrzeskie hemp variety. (Source: own photo).

Hemp concrete is gaining increasing popularity as a construction material due to its distinctive characteristics. Hempcrete is composed of a blend of lime, water, and hemp shives (Figure 1). These shives, originating from the hemp plant, are free from psychoactive compounds like THC [1]. Hemp shives are wooden parts of stems, which in the agricultural sector are treated as waste material after the plant has been harvested (Figure 2). Hempcrete is an eco-friendly material with numerous benefits that have caught the interest of architects and civil engineers [2].

One of the key characteristics of hempcrete is its excellent thermal insulation (0.087 W/m·K [3]), which enables the erection of exterior walls that meet current technical requirements (U ≤ 0.2 W/m^2^·K) for thermal insulation in Poland, without a need for additional insulation [4]. The material exhibits very low thermal conductivity, which means it effectively prevents heat loss during winter and reduces overheating in hot summers. As a result, hempcrete can substantially lower heating and cooling costs, as well as reduce greenhouse gas emissions from energy production [5,6]. In the case of conventional building materials, such as ceramic blocks by Wienerberger—Porotherm (e.g., 25 P + W)—or aerated concrete—Ytong PP4/0.6—the thermal conductivity coefficient typically reaches values of around 0.31 W/m·K or 0.21 W/m·K. These materials require an additional layer of insulation [7,8].

Another benefit of hempcrete is its positive impact on the indoor microclimate. The material is highly permeable to water vapor, allowing air to circulate freely and preventing moisture buildup. As a result, hempcrete can enhance indoor air quality, which is especially beneficial for individuals with allergies and respiratory conditions [9,10].

Hempcrete can be used as a soundproof material as it exhibits sound absorption ability. Hemp fibers absorb sound and dampen vibrations, which helps reduce noise. In order to achieve optimum insulating features, hempcrete must be designed and applied in a proper way, taking into account the proportions of the ingredients, the density of the mix, as well as the technical aspects of its application, such as ensuring proper filling around the frame or compacting the mix when using the formwork method. Hemp shives need to be uniformly distributed throughout the concrete mixture, and the thickness of the insulation layer should be appropriately chosen to meet soundproofing requirements [11].

Hemp concrete also shows low embedded energy, i.e., the amount of energy consumed during production and transportation of the material [12]. According to Florentin, the embedded energy of hemp concrete is 3.72 MJ/kg, which is 8% lower compared to autoclaved cellular concrete (4.04 MJ/kg) [13]. Due to these properties, the material is more sustainable and can contribute to lowering greenhouse gas emissions linked to building production [3,14].

The literature provides various examples of studies focused on determining the thermal conductivity coefficient of hemp concrete. For instance, the study by Abdellatef et al. presented tests on the thermal conductivity of hempcrete in different variants, with results varying from 0.087 to 0.101 W/m·K [15]. Another research group conducted laboratory tests on the thermal conductivity of hemp concrete from various mixtures and examined the effect of moisture content, which increases the thermal conductivity value. In this case, the obtained values ranged from 0.093 to 1.570 W/m·K [16].

Hemp concrete is valued for its insulating properties, but there is a lack of detailed and widely recognized research on its thermal conductivity under different conditions (e.g., different mix proportions, different climatic conditions). A body of research/experimental data needs to be developed until computational models can be created to simulate the properties of hemp concrete based on a given composition under given environmental conditions. Conducting thermal conductivity studies is crucial to accurately determine the energy efficiency of buildings constructed with hemp concrete. This, in turn, has a direct impact on the design of energy-efficient buildings and on meeting building standards. The condition also applies to other parameters such as compressive strength and flammability, among others.

Another gap in the science that our team plans to address is the lack of thorough investigation of long-term changes in these properties, for example due to the effect of moisture on thermal insulation. The lack of recognized and widely used norms and standards for hemp concrete hinders its widespread use in engineering practice. There is a need to develop test procedures that can be adopted at the national and international levels. Standardization allows for more widespread and trusted use of this material, facilitating its certification and integration into current building regulations.

This paper presents the results of a study on the thermal conductivity of hempcrete, composed of a lime–cement binder, as well as two other material variants based on the same binder but with metakaolin and FBC fly ash (fluidized bed combustion fly ash) added.

Such a modification is aimed at testing the changes in thermal conductivity coefficient and compressive strength after the addition of metakaolin and waste FBC fly ash, and assessing their potential use in eco-friendly buildings with enhanced durability. The application of FBC fly ash, which has hydraulic properties, is intended to initiate the development of a matrix and improve the durability of the material.

The main constituents of FBC ash are anhydrite (CaSO_4_), quartz (SiO_2_), lime (CaO), and/or portlandite (Ca(OH)_2_), and it may also contain unreacted calcite (CaCO_3_) [17]. The use of 20% FBC ash results in the greatest increase in compressive strength after 28 and 56 days. A higher content of FBCFA (>30%) reduces the compressive strength and rheological properties of concrete [18]. Another advantage of using fly ash is the effective management of this material, which is a byproduct of coal combustion in the power generation industry [19].

Metakaolin, on the other hand, acts as an active pozzolan containing a large amount of reactive silica and alumina, which reacts with calcium hydroxide formed during cement hydration. Secondary products such as secondary C-S-H gel (hydrated calcium silicate) and crystalline phases containing alumina, such as C_4_AH_13_, C_2_ASH_8_, and C_3_AH_6_, are formed, which fill the voids and strengthen the concrete structure [20]. This results in increased compressive strength, which is particularly noticeable after 28 days of curing. The results obtained with 15% by mass of metakaolin showed an improvement in compressive strength by 21.41% compared to the control sample (without mineral additives). This phenomenon is associated with the density of the microstructure and reduction in porosity caused by the pozzolanic reaction of metakaolin [21].

Another study of hemp concrete was conducted by Elfords and his team in 2022, where they also tested whether the use of metakaolin as a partial replacement for lime (0%, 10%, 20%) would improve the performance and durability of the material. The greatest improvement in compressive strength was observed with 20% metakaolin content. The control sample BCT2 (without mineral additives) achieved a compressive strength value of 1.52 MPa, while the sample BCT2MK (with 20% metakaolin) reached 1.71 MPa, representing an increase in strength by 12.5% [22].

The reduction of open porosity and modification of the pore system (towards smaller and less continuous pores) affects heat conduction and slows fire penetration deep into the structure, which may explain the observed improvement in fire resistance. According to Shewalul et al., the content of organic material (hemp shive) is responsible for smoldering and charring, but does not lead to significant fire spread. Moreover, the material neither drips nor emits flaming particles. The maximum temperature reached on the rear surface of the sample after 30 min of exposure remained below 250 °C for most configurations, where the critical threshold is typically considered to be above 300 °C—a level deemed hazardous for wooden and masonry structures [23].

In the case of FBC ash, the presence of active lime and sulphates can initiate reactions leading to the formation of secondary hydration products (e.g., ettringite and hydrated sulphate phases), which can also partially fill the pores and affect the microstructure. However, compared to metakaolin, these effects are less pronounced and depend on the curing conditions and moisture content of the material [24,25]. Materials with a higher content of reactive silica and aluminium show better thermal stability, not least due to the formation of amorphous phases that are resistant to rapid temperature changes [26]. It has also been considered that under elevated temperature conditions, C-A-S-H-type gels show greater stability than typical C-S-H, which also contributes to the improved fire resistance of the material containing metakaolin [27].

The first stage of work on modifications of the hempcrete composition involved the aforementioned verification of if the additive in the form of fly ash and metakaolin can be used without the deterioration of hempcrete’s thermal insulation properties.

Additional parameters tested were hempcrete flammability and resistance at different exposure times of specimens at 500 °C. The verification of these parameters will provide a better understanding of the material and indicate possibilities for its use in the building industry in general. The addition of waste to the composition of hempcrete also improves the eco-friendliness of building materials, reduces the use of natural raw materials in construction, and contributes to the implementation of the idea of a circular economy.

## 2. Materials and Methods

The hemp shives that were used in this research grow in eastern Poland and were supplied by the Podlaskie Konopie company (Dobrzyniowka, Poland). It is a variety of industrial hemp of the “whitethorn” species with a fraction of 5–25 mm. Hemp shives are a material extracted from the inner part of hemp stalks. They are lightweight, strong, and demonstrate high porosity, around 75.0% [28], which contributes to excellent thermal insulation properties, making them an ideal addition to eco-friendly building materials such as hemp concrete. In addition, hemp shives are biodegradable and renewable, which contributes to sustainability and environmental protection. The preparation of hemp shives involved storing them in a room with controlled temperature and humidity, i.e., 20 °C and relative humidity RH = 45%.

The binder used for testing consisted of hydrated lime and Portland cement (CEM I 42.5 R). Hydrated lime (Ca(OH)_2_), also known as slaked lime, is produced through the exothermic reaction of calcium oxide (CaO) with water. Due to its alkaline properties, it is widely used in construction, primarily as a component of mortar and plaster, improving their plasticity, adhesion, and microbiological resistance. Additionally, it is used in agriculture and the chemical industry as an acid neutralizing agent and pH stabilizer.

Portland cement CEM I 42.5 R, on the other hand, has a significantly higher compressive strength than hydrated lime and a rapid early strength gain, which is particularly important in the context of the pace of construction work and achieving the appropriate mechanical properties of the composite in a short time.

According to current literature data and the results of numerous life cycle analyses (LCA), the total CO_2_ emission in the production of hydrated lime (Ca(OH)_2_) is higher than that of Portland cement, which is influenced by factors such as: firing technology, type of fuel used, and the energy efficiency of the process, which can consume an average of 4500 MJ/t and release 1.1 t CO_2_/t Ca(OH)_2_ into the atmosphere [29]. In the case of the production of Portland cement CEM I, which consists of at least 95% Portland clinker, the production of 1.0 t of the product consumes approximately 3200–3600 MJ, which results in the emission of 0.92 t CO_2_/t clinker [30].

However, considering the ability of hydrated lime to undergo long-term carbonation (binding CO_2_ from the atmosphere) during the material’s service life (estimated to last over 50 years), its carbon footprint over the entire life cycle may be significantly lower than that of Portland cement. This process leads to a partial offsetting of the emissions from the production stage through formation of calcium carbonate (CaCO_3_) in the pores and on the surface of the material, reducing the final value of carbon dioxide emissions to 0.34 t CO_2_/t Ca(OH)_2_ [31].

According to Muhit et al. [32], more than half of the greenhouse gas emissions associated with hemp concrete depend on the type of binder used. Therefore, they emphasize that the choice of binder is crucial not only for the emission balance but also for the course of carbonation and sequestration processes. For this reason, they highlight the need for further research on optimizing the use of low-emission and environmentally friendly binders to fully understand their environmental impact.

The modification of hempcrete composition by introducing Portland cement and active mineral additives (such as fly ash or metakaolin) can significantly improve its durability and mechanical properties, but at the same time, it may reduce its biodegradability and recyclability. The presence of C-S-H and C-A-S-H mineral phases that strongly bond the cement matrix to the organic filler (hemp shive) makes it more difficult to recover and separate the components after the material’s service life has ended. In such a material system, the end-of-life (EoL) phase may involve the need for mechanical or chemical processing of the waste, which increases its environmental cost [33].

Therefore, a life cycle analysis (LCA) should include not only the production phase, but also the long-term effects associated with the use, dismantling, and disposal of hempcrete, which is essential for a full assessment of hempcrete sustainability [34].

The mineral additives used were metakaolin and fluidized-bed fly ash (FBC fly ash). Metakaolin is a highly reactive pozzolanic material obtained by calcining kaolinite at temperatures of 650–800 °C. It is used as an additive to concrete, increasing its strength and durability through pozzolanic reactions that create additional calcium silicate gel (CSH). Due to its fine-grained structure, metakaolin also reduces the porosity of concrete, improving its resistance to aggressive environments. On the other hand, fly ash from fluidized-bed combustion (FBC) boilers has a higher CaO content and lower SiO_2_ and Al_2_O_3_ contents compared to silica fly ash. Due to their pozzolanic reactivity, FBC ash can react with calcium hydroxide (Ca(OH)_2_) in concrete to form additional hydration products such as CSH and CASH, which improve the strength and durability of concrete. In addition, FBC ash contributes to reducing the porosity of concrete and improving its microstructure. The chemical composition of the components used is shown in Table 1.

Tests were carried out for three modified compositions of hempcrete. In total, 36 samples of 150 × 150 × 150 mm were prepared, i.e., 12 for each variant. Their composition varied by the content of mineral additives in a form of metakaolin and FBC fly ash as shown in Table 2.

**Figure 3:**
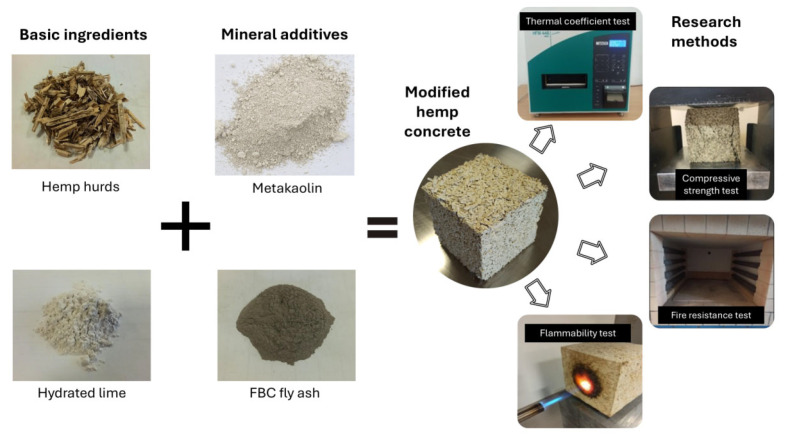
Diagram showing the ingredients used and the tests conducted on the hemp concrete samples made. (Source: authors’ own work).

The preparation of hemp concrete mixtures began with the dry mixing of the binding materials, followed by the addition of water and then hemp shives. The ingredients were mixed for 3 min before being placed in a metal mold and compacted using a lab shaker. The specimens were unmolded after 7 days of curing and then conditioned in air-dry conditions at 20 °C with continuous room ventilation for the next 90 days. The tests were conducted after 90 days to account for the use of a lime-based binder and the extended curing period of hempcrete. A graphical representation of the materials used and the tests conducted can be found in Figure 3.

The first batch of hempcrete tested, marked as “LC”, consisted of a two-ingredient binder in a form of hydrated lime and Portland cement CEM I 42.5 R and hemp shives. The second batch of concretes, marked as “LM”, contained hydrated lime, cement CEM I 42.5 R, and the addition of metakaolin in amount of 15% by mass. The LA hempcrete had a composition analogous to that of the LM concrete, except that FBC fly ash of 20% by mass was used instead of metakaolin.

The thermal conductivity of hempcrete was tested using the NETZSCH HFM 446 Lambda Small Eco-Line device (plate type) with a closed test chamber (Selb, Germany). The test was conducted in accordance with the PN-EN 12,667 standard, using the guarded hot plate method in a two-plate configuration [35]. Measurements were taken with a temperature difference of 10 K between the plates. Three cubic specimens of 150 × 150 × 150 mm were made for testing, from which 6 analytical samples were cut to obtain elements of 150 × 150 × 30 mm. Samples were taken from two opposite sides of the test piece. Results of thermal conductivity measurements for each composition were averaged.

**Figure 4:**
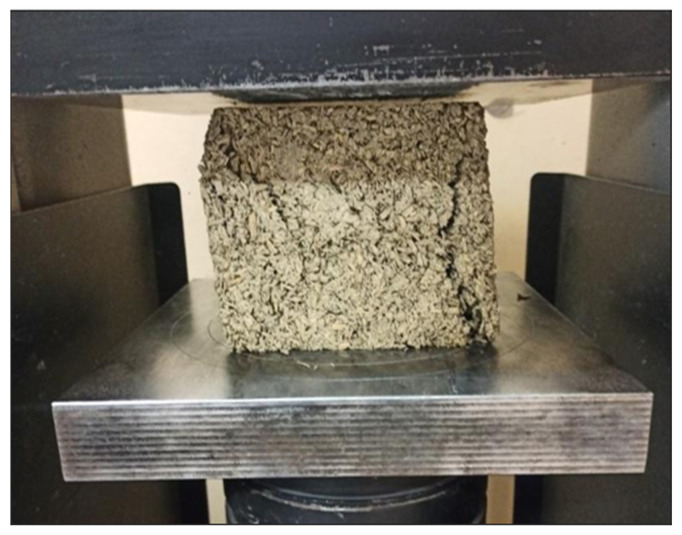
Photo of compressive strength test. (Source: own photo).

The compressive strength was tested on 5 test pieces using a hydraulic press manufactured by Controls (model C7600FR) (Figure 4) in line with the PN-EN 12390-3:2019-07 standard [36]. Results obtained for each of three batches of concretes tested were averaged.

**Figure 5:**
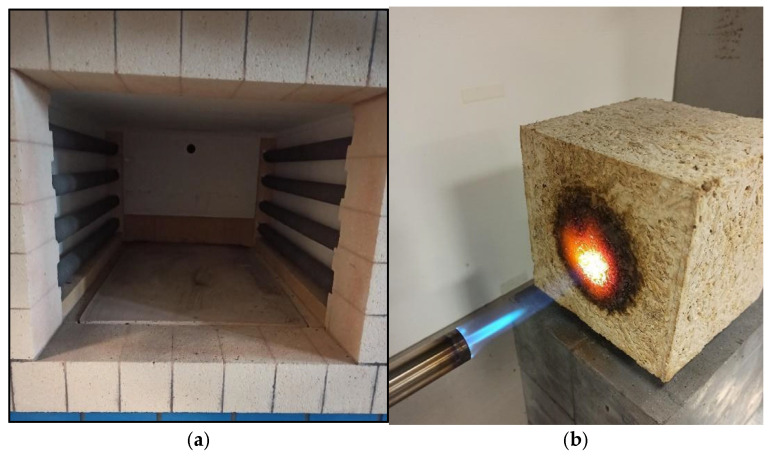
(**a**) Interior of a muffle furnace chamber; (**b**) flammability test with gas blow torch. (Source: own photo).

To test the flammability, the authors developed their own testing method, in which flammability was tested on three hempcrete specimens by their surface being directly exposed to a flame at 1100 °C (Figure 5b). The test lasted for 10 min, and the torch nozzle was positioned perpendicular to the sample surface at a distance of 100 mm. It was observed whether the fire would be extinguished or spread deeper into the specimen or on its surface when the fire exposure was interrupted. Each specimen was cut in half after cooling, and after removing loose material, the maximum distance to which the material was etched by the fire was measured. The test results provide a comparative value of the specimens’ reactions with different compositions (with metakaolin and fly ash FBC) to the fire source exposure, as well as an assessment of the potential impact of mineral additives on the fire-resistant properties.

While fire resistance testing was carried out in a muffle furnace type LHT 02/17 LB (Nabertherm GmbH, Lilienthal, Germany) for four specimens from each batch of concrete. The fire resistance testing in the muffle furnace was determined by visual analysis and the difference in mass of the specimens before and after exposure to high temperatures (Figure 5a). Two series of tests were carried out after 15 and 45 min of the specimen exposure to a temperature of 500 °C. The heating curve was linear, and the furnace reached the target temperature in about 20 min, after which it was maintained for a set exposure time (15 or 45 min). Cooling occurred naturally (furnace turned off, doors closed), without artificial forced air circulation. The specimens were removed after 3 h and weighed.

## 3. Results

**Figure 6:**
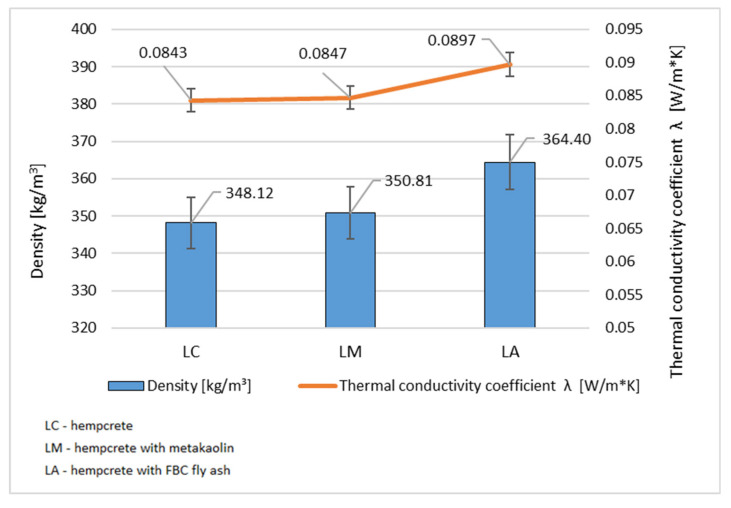
Dependence of thermal conductivity on density of hempcrete. (Source: authors’ own work).

All test specimens met the expected durability requirements and exhibited a consistent distribution of hemp shives throughout their cross sections. The LC specimen, which contained no mineral additives, had the lowest thermal conductivity coefficient. The specimen with metakaolin showed a slightly higher thermal conductivity, with a 1.51% increase compared to the LC specimen, despite having a similar density. On the other hand, the LA composite (containing FBC fly ash) exhibited the highest thermal conductivity. This additive appears to enhance the sealing of the microstructure, as evidenced by the increased thermal conductivity and compressive strength, which in turn decreased the thermal insulation properties of the composite. The relationship between thermal conductivity and hempcrete density is illustrated in Figure 6.

**Figure 7:**
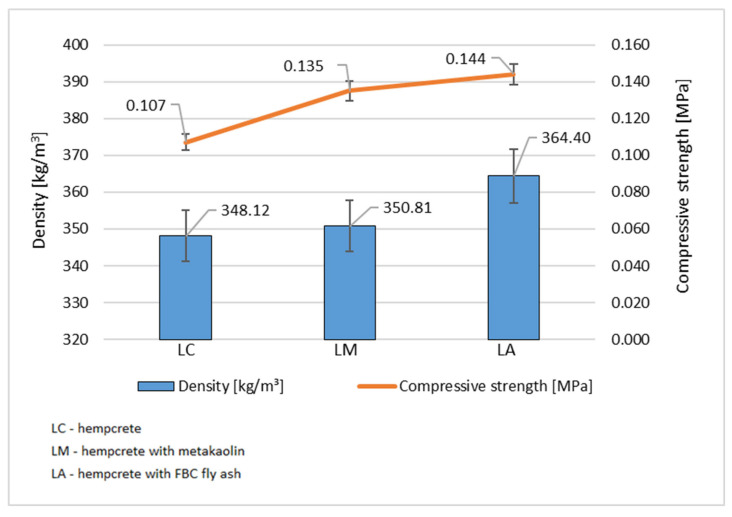
Dependence of hempcrete compressive strength on its density. (Source: authors’ own work).

The compressive strength test carried out showed that no composition reached a value above 0.15 MPa (Figure 7). The obtained results confirmed that the material of modified hempcrete being tested, regardless of the mineral additive used, can fulfil only a task of filling the partition due to its very good thermal insulation properties. Application of metakaolin in the LM specimen increased the hempcrete compressive strength compared to the LC specimen by 26%. LA hempcrete with the addition of FBC fly ash showed 35% higher compressive strength values than LC concrete, which may also be partly due to the increased density of that composition and partly due to formation of an additional amount of a C-S-H phase in the concrete matrix as confirmed by tests by other research teams. This is due to the fact that FBC ashes contain silicon oxides (SiO_2_), aluminum (Al_2_O_3_), calcium (CaO), and iron (Fe_2_O_3_), which are key in the hydration processes of cement and the formation of additional C-S-H and C-A-S-H products, as well as, e.g., hydroxyaluminum or ettringite. Metakaolin, on the other hand, is a highly reactive pozzolanic material obtained from kaolinite by its calcination at temperatures of 650–800 °C. The main ingredients of metakaolin are SiO_2_ (silica) and Al_2_O_3_ (aluminum), making metakaolin highly reactive in the presence of calcium hydroxide to form additional C-S-H and C-A-S-H products, as well as hydroxyaluminum, hydrotalcite, C-A-H phase, or ettringite [37,38].

Flammability tests demonstrated the resistance of all hempcrete compositions to the spread of fire. After the torch was switched off, each sample continued to glow for about three minutes, but slowly cooled down until it extinguished itself (Figure 5b). The measured maximum depth of fire etching of each sample is shown in Table 3.

**Figure 8:**
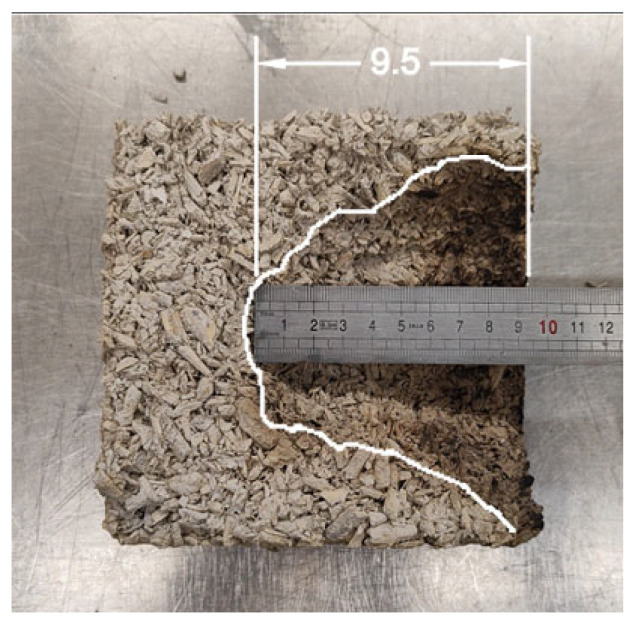
Depth of material burnout after hemp concrete flammability test. (Source: own photo).

The flammability test showed increased resistance of the samples with mineral additives to disintegration of the structure as shown by the results of the depth to which the fire reached. The unmodified hemp concrete was burned to a depth of 95.0 mm (Figure 8). The sample containing 15% metakaolin additive showed the best properties. Exposure to flame and high temperature disintegrated the structure of the LM material to a depth of only 45.0 mm. A very similar result (50.0 mm) was achieved by the composition of LA hemp concrete with a 20% addition of FBC fly ash. Clearly, the conclusions of this study indicate that the use of mineral additives almost doubles the flame resistance of hemp concrete.

The averaged results of the mass difference of each hempcrete composition after testing in the muffle furnace (Figure 5a) are shown in Table 4 and Table 5.

Observations showed that the LC composite without mineral additives demonstrated the highest mass loss in both fire resistance tests, i.e., 15 and 45 min exposure of the composite to high temperature (500 °C). This significant mass loss by 21.67% and 30.98%, respectively, indicates that the hempcrete with the modified composition in the LC sample may have a lower resistance to high temperatures, which is an important factor for applications in civil engineering structures.

The mass of the LM sample with metakaolin in the 15 min exposure test at the temperature of 500 °C decreased by 13.71%. While, after 45 min in the chamber, it decreased by 26.22%, which is the smallest mass loss among the concretes tested. In addition, a mineral additive in the form of metakaolin contributed to a significant improvement in the resistance of this concrete to high temperatures up to 500 °C.

**Figure 9:**
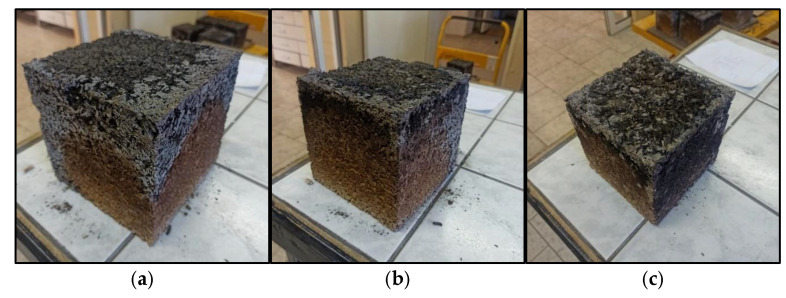
Sample after fire resistance test in muffle furnace for 45 min: (**a**) LC (hempcrete); (**b**) LM (hempcrete with metakaolin); (**c**) LA (hempcrete with FBC fly ash). (Source: own photo).

The LA composite also showed better resistance to high temperature (500 °C) compared to the LC sample due to a mass loss of 15.86% in the 15 min fire resistance test and 29.94% in the 45 min fire resistance test. This gives a clear improvement in the fire resistance of the LA composite compared to the hempcrete with unmodified composition. Figure 9 shows a condition of specimens after 45 min fire resistance testing.

## 4. Discussion

**Figure 10:**
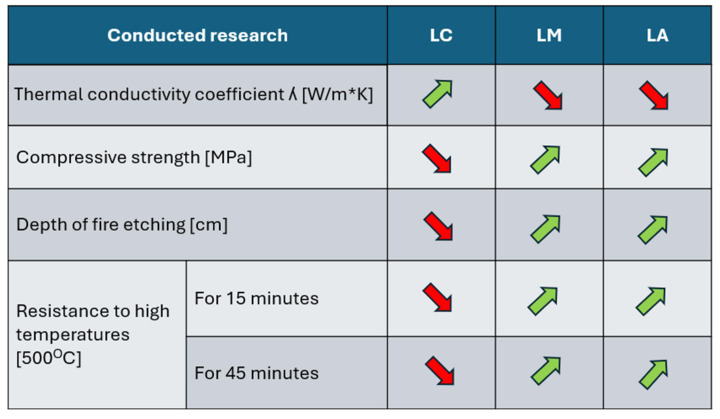
Diagram of changes in material properties under the influence of mineral additives. Green color indicates improvement of properties. Red color means deterioration of properties. (Source: authors’ own work).

The compilation of the results from the experiments made it possible to make an illustrative summary of how mineral additives affect the properties of hemp concrete (Figure 10). The summary shows the improvement of strength and resistance parameters at the expense of deterioration of the thermal conductivity coefficient.

The conducted tests confirmed a potential use of metakaolin and FBC fly ash as additives. These materials did not interfere with the specimen preparation process or the anticipated curing duration. An important conclusion is that the use of metakaolin increased the hempcrete compressive strength. A similar relation was demonstrated by the team of A. Borçato during their research on hempcrete composition with different proportions of metakaolin added [39]. However, hempcrete is considered a non-structural (non-load-bearing material), primarily intended for insulating and filling applications, such as in wall partitions, thermal insulation layers, partition walls, or passive enclosures. The main reason for the increased thermal conductivity of hempcrete after the addition of metakaolin and FBC fly ash is the increase in the density of specimens. All values tested are within a range well below ranges for traditional building materials, so hemp concrete is a good insulator and can be effectively used for thermal insulation. Similar results were obtained by Baib Jirgensone at the level of ʎ = 0.076 W/(m·K), which also indicates the very good thermal insulation properties of hempcrete [40].

The use of hemp shives as an additive to cement-based composites significantly affects the physical, mechanical, and thermal properties of the resulting material. Hemp shives, as lightweight, porous organic materials with low thermal conductivity, contribute to the improved thermal insulation of hemp concrete. However, their presence also leads to a reduction in bulk density and a decrease in mechanical strength, which poses a significant challenge in the design and practical application of such composites.

It has been demonstrated in the literature that increasing the proportion of lignocellulosic materials, such as hemp shives, in concrete mixtures can reduce thermal conductivity by as much as 30–60% compared to conventional concrete [5,41]. At the same time, it has been found that compressive strength may be reduced to a level of 0.5–5 MPa, depending on the type of binder used, the water-to-binder ratio (*w*/*b*), and the degree of compaction [42]. It therefore becomes necessary to seek a compromise between improving thermal properties and ensuring an acceptable level of load-bearing capacity, especially in the context of using the material in wall partitions.

This study analyses the influence of hemp shive content on the thermal conductivity and compressive strength of the mixes. The results obtained confirm that increasing the amount of organic material leads to a systematic decrease in thermal conductivity, which is beneficial from the perspective of the energy efficiency of building partitions. However, a significant reduction in mechanical strength was observed, which greatly limits the possibility of using such mixes in structural elements and directs their application primarily towards non-structural solutions, such as wall infill.

Potential directions for optimizing the material composition include the application of hybrid binder systems (e.g., lime–pozzolanic or cement–fly ash mixtures), modifiers that enhance adhesion between the organic phase and the matrix, and control of the particle size distribution of the hemp shives to achieve better compaction characteristics. The aim of these efforts is to develop a material with balanced properties, offering both satisfactory thermal insulation and sufficient mechanical strength for use in building applications.

**Figure 11:**
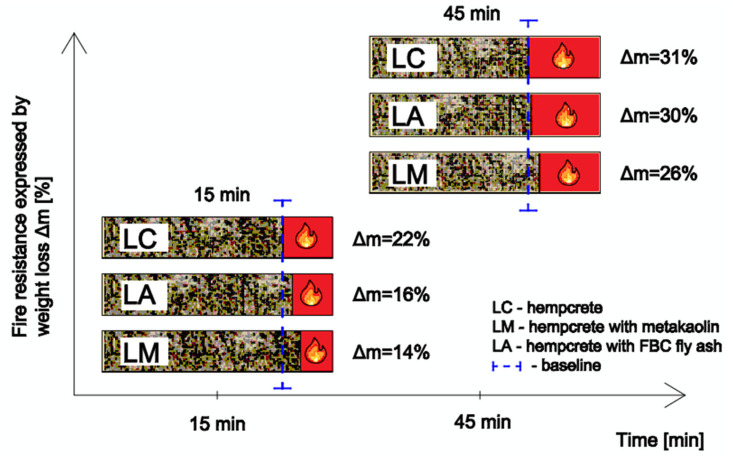
Weight loss of hemp concrete samples (LC, LA, LM) after fire resistance test in muffle furnace for 15 and 45 min. (Source: authors’ own work).

An important aspect of hempcrete use is also its fire safety, which has been initially confirmed and the flammability test results clearly showed the resistance of all compositions to the spread of fire (Figure 11).

However, the results of fire resistance tests suggest that the composition of hempcrete can have a significant impact on its ability to maintain strength and integrity at high temperatures. Optimization of the concrete composition, including proper use of hemp-based materials, can be crucial to ensure the desired resistance to high temperatures. A similar test was conducted by a research team from Australia led by Shanaka Kristombu Baduge, who determined a 20% mass loss in a test at 300 °C [43]. Subsequent tests of hemp concrete conducted by the team of Shewalul also showed that the material demonstrated a high resistance to the spread of fire and also maintained integrity of the partition even after two hours of exposure to direct fire, in line with ISO 834 [23,44].

The lowest mass loss was demonstrated by the LM hempcrete composition with the mineral additive in the form of metakaolin, which significantly improved durability of the material. Also, the addition of fly ash in LA samples showed the improvement in hempcrete properties, although not to the same extent as the composition containing metakaolin, as can be seen in Table 3 and Table 4.

When analyzing the microstructural mechanisms affecting the properties of hemp concrete, special attention should be paid to the type of binder applied. The use of lime-based or lime–cement binders in the presence of industrial hemp fibers results in significant differences in the microstructure compared to conventional concretes.

One of the key processes is the formation of a C-S-H (calcium silicate) gel, the main component responsible for the material strength. Although hemp fibers may locally delay hydration, studies suggest that the cellulose and hemicellulose present in their structure have the ability to bind calcium ions, which can promote the local formation of C-S-H gel. Moreover, in the presence of pozzolans (e.g., fly ash), the presence of lignin and pectin can initiate the formation of additional phases such as C-A-S-H gel (calcium–aluminum–silicate), which improves the microstructure of the paste [45].

The high porosity of hemp concrete is on the one hand an advantage (good thermal insulation), but on the other hand can lead to reduced durability. A phenomenon that improves the impermeability of the structure is the secondary sealing of pores through carbonation, i.e., the reaction of calcium hydroxide with CO_2_ from the air. A product of this reaction is calcium carbonate (CaCO_3_), which is deposited in micro- and mesopores, effectively reducing water permeability and the migration of aggressive ions [42,46]. Carbonation can also occur in the fiber-adjoining zones, leading to local sealing and an increase in the resistance of the concrete to environmental factors [47].

The interfacial transition zone (ITZ) plays a crucial role in the structure of hempcrete, forming at the interface between hemp fibers and the paste. Its quality depends on many factors, including the water absorption of the fibers, which can draw water from the paste and cause local disruptions in the hydration process. Impregnation of the fibers with lime or other adhesion-enhancing agents helps to reduce these effects and improve the cohesion of the ITZ [48]. Microscopic testing (e.g., SEM) has shown that a well-developed ITZ increases the material’s resistance to micro-cracking and enhances its mechanical integrity [49].

Due to the organic nature of the filler, a significant microstructural threat comes from alkaline reactions between the binder and lignocellulosic components. The high pH of the cement matrix can lead to fiber structure degradation, resulting in a decreased material strength. The use of pozzolans, such as metakaolin or fly ash, allows a partial reduction in the pH value of the environment and slows down the rate of degradation. Furthermore, mineralization of the fibers can occur over time through the deposition of hydration products in their structure, increasing their durability and resistance to biodegradation [50].

All these phenomena occurring within the microstructure have a direct impact on the macroscopic properties of hempcrete, such as compressive strength, resistance to freeze–thaw cycles, and thermal conductivity. Optimization of the ITZ and pore sealing leads to increased resistance to water exposure and dimensional stability of the material over its long-term use [51].

Based on the results obtained, it can be assumed that metakaolin and fluidized bed ash (FBC) affect hempcrete microstructure and durability through different physical and chemical mechanisms.

In the case of metakaolin, its high content of reactive silica and alumina may promote the intensification of pozzolanic reactions, leading to the formation of C-A-S-H gel. Formation of this phase likely improves the impermeability of the interfacial transition zone (ITZ) between the hemp fibers and the matrix, limiting the development of micropores and enhancing the cohesion of the composite. Additionally, the reduction of pH value due to the binding of free lime may reduce the risk of degradation of the lignocellulosic components of the fibers.

On the other hand, in the case of FBC ash, the presence of active lime and sulphates may initiate other secondary reactions, such as the formation of hydrated phases and potential deposition of hydration products on the surface of hemp fibers. It can be assumed that this leads to the partial mineralization of fibers, which increases their resistance to biodegradation and reduces their sensitivity to the alkaline environment. At the same time, these secondary products can seal the internal structure of the composite, reducing permeability and improving its durability.

Although these mechanisms require further confirmation through microstructural testing (e.g., SEM-EDS, XRD), the obtained results suggest that both additives may modify hempcrete properties in a way that is significantly different from the effect of traditional pozzolans. This indicates a potentially innovative direction for further research and the possibility of designing composites with optimized structure and durability using alternative mineral additives.

## 5. Conclusions

Based on the conducted experimental studies, the following conclusions were drawn:

(1) Mineral additives in the form of metakaolin and FBC fly ash cause deterioration of the thermal conductivity coefficient by 0.5% when metakaolin is used at 15% by mass of the binder, and by 6% when FBC fly ash is used at 20% by mass of binder.
(2) The strength of hempcrete does not allow its use to carry the loads of the structure. Nevertheless, the use of mineral additives resulted in improved compressive strength values of LM samples by 26% (0.135 MPa) and LA samples by 35% (0.144 MPa) compared to the unmodified formulation of LC (0.107 MPa).
(3) Hemp concrete is a self-extinguishing material.
(4) The fire resistance testing at 500 °C for 15 min has showed that application of mineral additives clearly improves durability of the material. The composition modified with addition of metakaolin has achieved a lower mass loss by approx. 58% relative to the LC composite, while addition of FBC fly ash (LA) leads to a mass loss by ca. 37%. However, after a period of 45 min under these conditions, differences between concretes without mineral additives and the modified concretes were not significant. The mass loss of the hemp concrete with addition of metakaolin was about 18% relative to the LC samples, while addition of FBC fly ash resulted in a mass loss by ca. 3.5% relative to the composite without mineral additives.

The obtained results confirm the possibility for the modification of hempcrete properties through the proper selection of mineral additives; however, further microstructural analyses and durability assessments over a longer time horizon are necessary.

## Figures and Tables

**Table 1:** Chemical composition of hydrated lime, Portland cement CEM I 42.5 R, metakaolin, and FBC fly ash by X-ray fluorescence (XRF).

Number	Parameter Determined	Result [%]			
		Portland Cement CEM I 42.5 R	Hydrated Lime	FBC Fly Ash	Metakaolin
1	CaO	65.21	97.80	16.46	1.20
2	SiO_2_	22.17	1.03	38.63	54.90
3	Fe_2_O_3_	3.85	0.30	7.84	1.30
4	SO_3_	3.29	0.17	7.18	-
5	Al_2_O_3_	3.09	-	22.42	35.30
6	K_2_O	0.84	0.06	2.98	2.70
7	MgO	0.83	0.44	1.77	0.40
8	TiO_2_	0.27	-	1.53	-
9	Na_2_O	-	-	1.01	-

**Table 2:** Material composition of hemp concrete samples.

Designation	Binder [kg/m^3^]				Filler [kg/m^3^]	Water [mL]	Filler to Binder Mass Ratio
	Hydrated Lime	Portland Cement CEM I 42.5 R	FBC Fly Ash	Metakaolin	Hemp Shives		
LC	218	54	-	-	136	2800	2.0
LM	177	54	-	41	136	3000	2.0
LA	163	54	54	-	136	3400	2.0

**Table 3:** Maximum depth of material etching by fire.

Designation	Depth of Burnout [mm]
LC	95.0
LM	45.0
LA	50.0

**Table 4:** Mass difference of hempcrete specimens cured for 90 days before and after 15 min of sample conditioning at 500 °C.

Designation	Specimen Mass Before Fire Resistance Testing [g]	Specimen Mass After Fire Resistance Testing [g]	Δm [%]
LC	1569.2	1229.1	21.67
LM	1515.5	1307.7	13.71
LA	1571.1	1321.9	15.86

**Table 5:** Mass difference of hempcrete specimens cured for 90 days before and after 45 min of sample conditioning at 500 °C.

Designation	Specimen Mass Before Fire Resistance Testing [g]	Specimen Mass After Fire Resistance Testing [g]	Δm [%]
LC	1452.2	1002.3	30.98
LM	1522.4	1123.2	26.22
LA	1890.1	1324.2	29.94

## Data Availability

The research data analyzed and generated during this study are available upon request from the corresponding author (Igor Klementowski, i.klementowski@po.edu.pl). Access to the data will be granted on a case-by-case basis in compliance with applicable privacy regulations and data protection laws.
